# Preoperative multiclass classification of thymic mass lesions based on radiomics and machine learning

**DOI:** 10.1186/s40644-025-00839-3

**Published:** 2025-03-06

**Authors:** Yan Zhu, Li Wang, Aichao Ruan, Zhiyu Peng, Zhenzhong Zhang

**Affiliations:** 1 Radiologic Department, The Affiliated Huaian No. 1 People’s Hospital of Nanjing Medical University, Huai’an, PR China; 2 General surgery Department, Huai’an Cancer Hospital, Huai’an, PR China; 3 Thoracic Surgical Department, The Affiliated Huaian No. 1 People’s Hospital of Nanjing Medical University, Huai’an, PR China; 4 Thoracic Surgery Department, West China Hospital, Sichuan University, Chengdu, PR China

**Keywords:** Thymic mass lesions, Radiomics, Classifier model, Early diagnosis

## Abstract

**Background:**

Apart from rare cases such as lymphomas, germ cell tumors, neuroendocrine neoplasms, and thymic hyperplasia, thymic mass lesions (TMLs) are typically categorized into cysts, and thymomas. However, the classification results cannot be determined in advance and can only be confirmed through postoperative pathology. Therefore, the objective of this study is to rely on clinical parameters and radiomic features extracted from chest computed tomography (CT) scans to facilitate the preoperative classification of TMLs. The model development specifically focused on thymic cysts and thymomas, as these are the most commonly encountered anterior mediastinal tumors in clinical practice.

**Materials and methods:**

This retrospective study included 400 participants from 3 hospitals between September 2017 and September 2024 due to TMLs. The participants were classified into 7 groups based on the ultimately confirmed etiology: thymic cysts and thymomas, including types A, AB, B1, B2, B3, and C. All participants underwent contrast-enhanced chest CT scans, with senior radiologists delineating regions of interest to extract radiomic features. Additionally, the participants’ ages were also collected as clinical parameters for analysis. The participants were randomly allocated into a training set and a validation set at a 7:3 ratio. A classifier models were developed using the data from the training set, and their performances were evaluated on the validation set.

**Results:**

The model exhibited good classification performance with accuracies of 0.8547.

**Conclusion:**

The model can assist in early diagnosis and the development of personalized treatment strategies for patients with TMLs.

**Supplementary Information:**

The online version contains supplementary material available at 10.1186/s40644-025-00839-3.

## Background

### Introduction

The thymus, located in the anterior mediastinum, plays a crucial role in early immune development by generating a diverse T cell repertoire essential for immune self-tolerance and defense [1]. Recent advances in high-resolution imaging, radiomics, single-cell omics, and organoid cultures have greatly enhanced our understanding of thymic structure, cellular dynamics, T cell development, and thymic mass lesions (TMLs), which encompass a range of both benign and malignant pathologies [2, 3]. Common TMLs include:

- Thymomas: The most common primary tumor of the thymus, classified according to the World Health Organization classification system into types A, AB, B1, B2, and B3 [4]. Thymomas exhibit a range of biological behavior, and are often associated with autoimmune diseases such as myasthenia gravis.
- Thymic Carcinomas (thymomas Type C): These are highly malignant tumor that grows rapidly and are prone to local invasion and distant metastasis.
- Thymic Cysts: Generally benign, these are often asymptomatic and discovered incidentally. However, thymic cysts may occasionally be associated with malignant lesions, such as thymic carcinoma or lymphoma, although this is relatively rare.
- Others: This category includes lymphomas, germ cell tumors, neuroendocrine neoplasms, and thymic hyperplasia [5].

TMLs are often asymptomatic in the early stages and may be incidentally found during physical examinations or imaging studies. Some patients may experience symptoms such as chest pain, cough, or shortness of breath, especially when the mass is large. Additionally, thymoma patients frequently present with autoimmune diseases, most commonly myasthenia gravis [6].

### Rationale and knowledge gap

Due to the diverse imaging presentations of TMLs and the overlap between different lesion types, accurate preoperative classification poses a significant challenge. Traditionally, definitive diagnosis relies on postoperative pathology; however, precise preoperative classification is essential for optimal treatment planning and prognostic assessment. Studies suggest that limited resection may be safe and potentially beneficial for patients with thymic cysts or early-stage, non-myasthenic thymomas [7–9]. In recent years, the application of radiomics and machine learning in tumor diagnosis has introduced new approaches for non-invasive preoperative assessment. By extracting quantitative features from imaging modalities such as chest computed tomography (CT), Magnetic Resonance Imaging, and Positron Emission Tomography-Computed Tomography and integrating them with clinical parameters, machine learning models hold promise for achieving more accurate preoperative classifications of TMLs, thereby optimizing treatment decisions [10–12]. Furthermore, some studies report that predictive biomarkers may serve as important biological markers to differentiate between thymomas and thymic cysts [13]. Existing studies, however, have largely been limited to binary classification in predefined scenarios, such as distinguishing between benign and malignant lesions, differentiating cysts from solid tumors, and assessing surgical risk [14–16]. A more detailed approach to multiclass classification has yet to be implemented.

### Objective

To enable early diagnosis and tailor personalized treatment strategies, we aim to construct a multiclass classifier model for TMLs. This study focuses on thymic cysts and thymomas, which are the most common and well-studied anterior mediastinal masses. Many similar studies have adopted comparable case selection criteria, ensuring that our research remains focused and comparable to existing literature. Moreover, during the data collection process, we found that cases of lymphomas, germ cell tumors, neuroendocrine neoplasms, and thymic hyperplasia were relatively rare. To ensure the quality and reliability of the model training, we decided to exclude these conditions and focus exclusively on thymic cysts and thymomas, including types A, AB, B1, B2, B3, and C.

## Methods

### Participants, groupings, and training/validation set split

The study involved 400 participants from three institutions: The Affiliated Huai’an No.1 People’s Hospital of Nanjing Medical University (174 cases), West China Hospital (223 cases), and Huai’an Cancer Hospital (3 cases), between September 2017 and September 2024. This study was conducted in accordance with the Declaration of Helsinki (as revised in 2024). The study was approved by the Institutional Review Board and Ethics Review Committee at The Affiliated Huai’an No.1 People’s Hospital, Nanjing Medical University (KY-2024-365-01), approved by the Ethics Committee on Biomedical Research, West China Hospital, Sichuan University (No. 2024 − 1511), approved by the Ethics Review Committee, Huai’an Cancer Hospital (No. 2024056). And informed consent for this retrospective analysis was obtained from each individual. Among the participants, there were 182 males and 218 females.

#### Inclusion criteria

- Participants initially diagnosed with TMLs by contrast-enhanced CT scan, and with discernible regions of interest (ROIs) on CT images;
- Participants whose diagnosis was finally confirmed through postoperative pathology, and their diagnoses fell into one of the following categories: Class_0 (thymic cysts), Class_1 (thymomas type A), Class_2 (thymomas type AB), Class_3 (thymomas type B1), Class_4 (thymomas type B2), Class_5 (thymomas type B3), or Class_6 (thymomas Type C).

#### Exclusion criteria

- Participants who received other treatments, such as chemotherapy or radiotherapy, before undergoing CT scan.
- Postoperative pathology of the participant indicated a mixture, such as of B1 and B2 or B2 and B3 types.

**Fig. 1:**
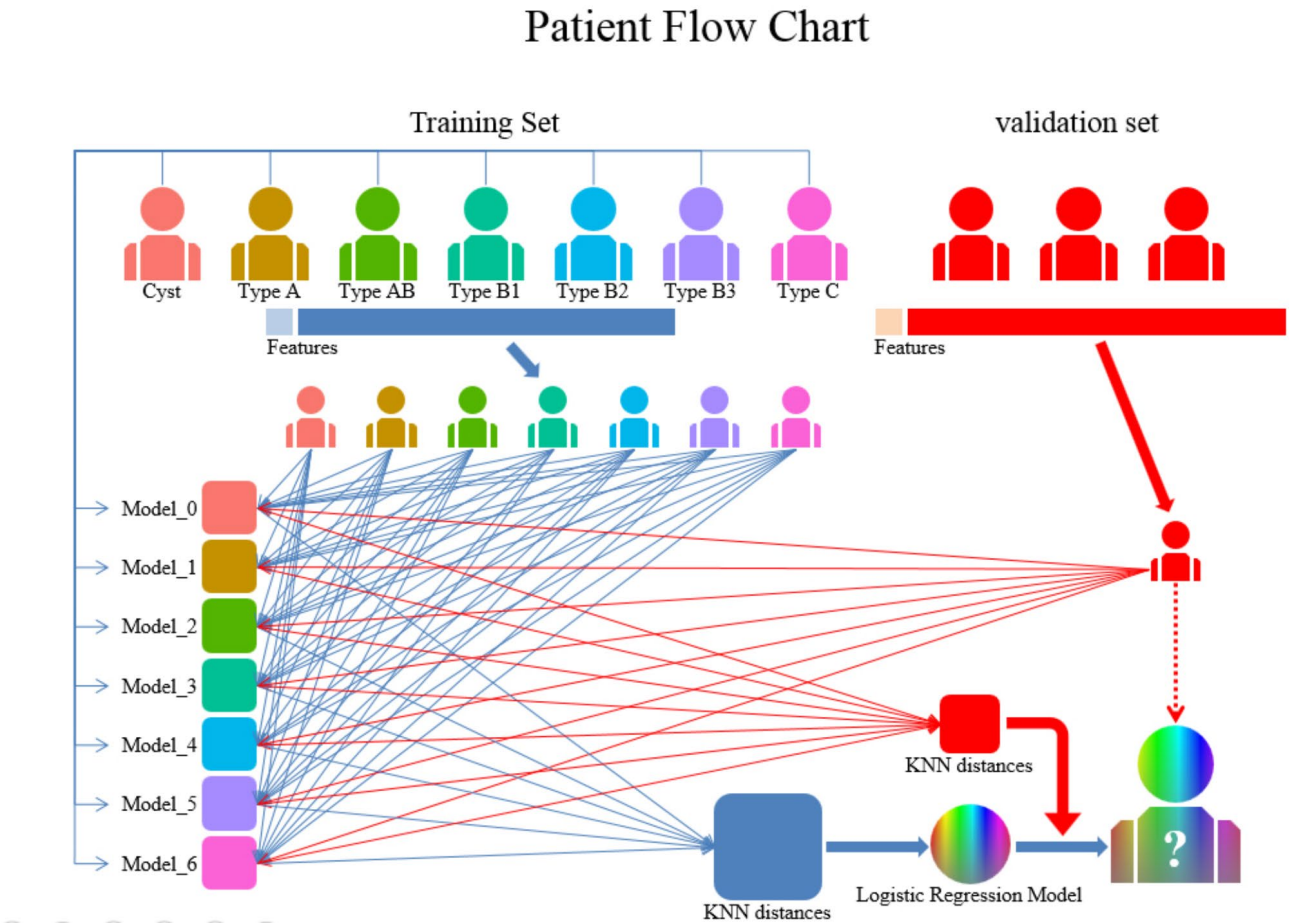
The patient flow chart. This figure shows the patient flow chart: Participants are randomly allocated to the training set and validation set in a 7:3 ratio. Using participants of specific categories from the training set as a reference, the KNN distances are calculated. After obtaining the KNN distances for all individuals across all categories, a logistic regression model is constructed, and the predicted outcomes for participants in the validation set are calculated

Out of the 400 participants, 122 were with thymic cysts, 15 were with thymomas type A, 78 were with thymomas type AB, 41 were with thymomas type B1, 60 were with thymomas type B2, 28 were with thymomas type B3, and 56 were with thymomas type C. The general clinical information of the participants is presented in Table 1. These participants were randomly allocated into a training set and a validation set using the “*caret*” package in R, with a split ratio of 7 to 3. The patient flow chart is shown in Fig. 1.

**Table 1:** The basic information of paitients

	*n*	age (mean (SD))	sex = male (%)
Overall	400	53.27 (11.51)	182 (45.5)
Cyst	122	53.55 (10.59)	43 (35.2)
Type A	15	58.93 (13.20)	11 (73.3)
Type AB	78	53.82 (11.39)	34 (43.6)
Type B1	41	51.02 (14.89)	21 (51.2)
Type B2	60	50.72 (10.30)	24 (40.0)
Type B3	28	50.07 (11.45)	18 (64.3)
Type C	56	56.38 (10.65)	31 (55.4)
p		0.022	0.008

### Software used

The software utilized in this study included the following: “ITK_SNAP” (version 3.4; http://www.itksnap.org), “pyradiomics” (https://pyradiomics.readthedocs.io), “Jupyter” (version 5.6.0; http://jupyter.org), “Anaconda Navigator” (version 1.9.2; https://www.anaconda.com) and “R Studio” (version 1.4.1106; www.rstudio.com).

### Chest CT scanning and radiomic features

Prior to the operation, each participant underwent a chest CT scan by a 64-row CT scanner (SOMATOM Definition, Siemens Healthcare, Forchheim, Germany). The CT scan parameters were as follows: tube voltage set at 120 kV, tube current modulation with automatic exposure control, collimation of 64 × 0.6 mm, rotation speed of 0.5 s/rotation, slice thickness of 1.0 mm, spiral pitch of 0.6 mm, and image reconstruction with 5 mm slice thickness. The resulting images of all participants were stored in the Digital Imaging and Communications in Medicine (DICOM) format.

### Extraction of radiomic features

The DICOM files were downloaded and uploaded to ITK-SNAP for processing. The Curve-Based Contrast Adjustment Settings are as follows: Control Point 1 (ID: Id1) is set at a gray level of -400 with an output contrast of 0, meaning that brightness is completely suppressed at this value. Control Point 2 (ID: Id2) is set at a gray level of 400 with an output contrast very close to the maximum (0.999), thereby enhancing the contrast and brightness in the desired range (-400 to 400). Control Point 3 (ID: Id3) is set at a gray level of 3000 with an output contrast reaching the maximum value (1.000), indicating that brightness values from 400 to 3000 are ignored. These settings are designed to suppress brightness at lower gray levels (e.g., -400), enhance contrast and brightness in the desired range, and ignore higher gray values (e.g., those greater than 400), effectively highlighting the target structures and reducing background noise. The ROIs were delineated according to the following criteria:

- The delineation process was completed under the joint supervision of a senior radiologist and a senior thoracic surgeon.
- The ROIs were restricted to remain within the boundaries of the tumor.
- A portion of the tumor was selected as the sampled region, but the selection was random.

The extraction of radiomic features from the ROIs was performed using “*pyradiomics*,” a package in Python, within the “Jupyter” environment, which is a component of the “Anaconda Navigator” software. A total of 129 radiomic features were extracted from each sample, and 19 columns of text information were disregarded. Additionally, since the shapes of the sampled regions were artificially determined and independent of the nature of the ROIs, all 14 radiomic features related to the shape were excluded. The inter-group differences of the radiomic features were calculated using the “*tableone*”package in R. Features with *p* < 0.05 were retained, resulting in 72 features for further analysis.

In addition, the participants’ ages were also collected as clinical parameters for analysis. The *p*-values of the radiomic features and age can be found in the supplementary files.

### Construction of base classifiers

**Fig. 2:**
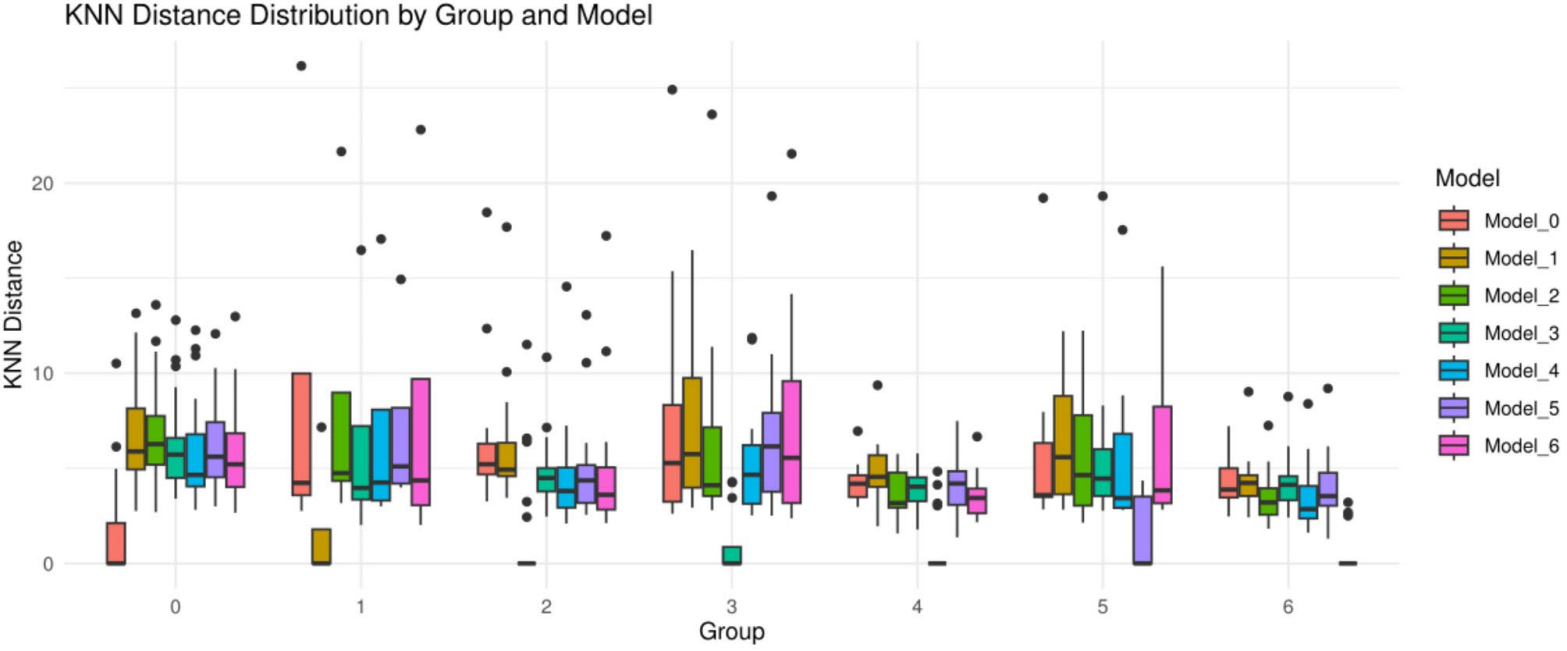
KNN Distance Distribution by Group and Model. This figure shows box plots of KNN distances for participants of different categories (0–6) in the validation set, distinguished by different colors for each model. For example, participants in group_0 have significantly lower KNN distances in model_0 compared to their results in other models. This demonstrates the significant contribution of KNN distances in classification decisions

The ages of all participants and the 72 radiomic features have been normalized and used for analysis. The K-Nearest Neighbors (KNN) algorithm was employed to build base classifiers. A total of seven models were constructed: Model_0 for thymic cysts, Model_1 for thymomas type A, Model_2 for thymomas type AB, Model_3 for thymomas type B1, Model_4 for thymomas type B2, Model_5 for thymomas type B3, and Model_6 for thymomas type C. Taking Model_0 as an example, all participants in the training set with the true category of thymic cysts were used as the reference set for the KNN algorithm. The KNN distances between all participants in the training set and the reference set were calculated with K = 1 using the “*KNN*”package in R. Subsequently, the KNN distances between the participants in the validation set and the reference set were also computed. These distances were then saved for further analysis. Similarly, the KNN distances for the other models were also calculated and saved. The boxplot based on the validation set, titled “KNN Distance Distribution by Group and Model,” is presented in Fig. 2, created using the “*ggplot2*”package in R.

### Construction of the meta-classifier

After the previous calculations, we obtained a matrix of KNN distances containing 400 rows and 7 columns. The original partitioning scheme is maintained, with 70% of the data allocated to the training set and the remaining 30% to the validation set. A multiclass logistic regression model was constructed on the training set using the “*caret*”package in R, with the method set to “multinom” and trainControl configured for 6-fold cross-validation. The KNN distances of participants in the validation set were used to evaluate the performance of this multiclass logistic regression model. We provide all the de-identified raw data and R code in the supplementary materials of the paper.

## Results

### Model performance

**Fig. 3:**
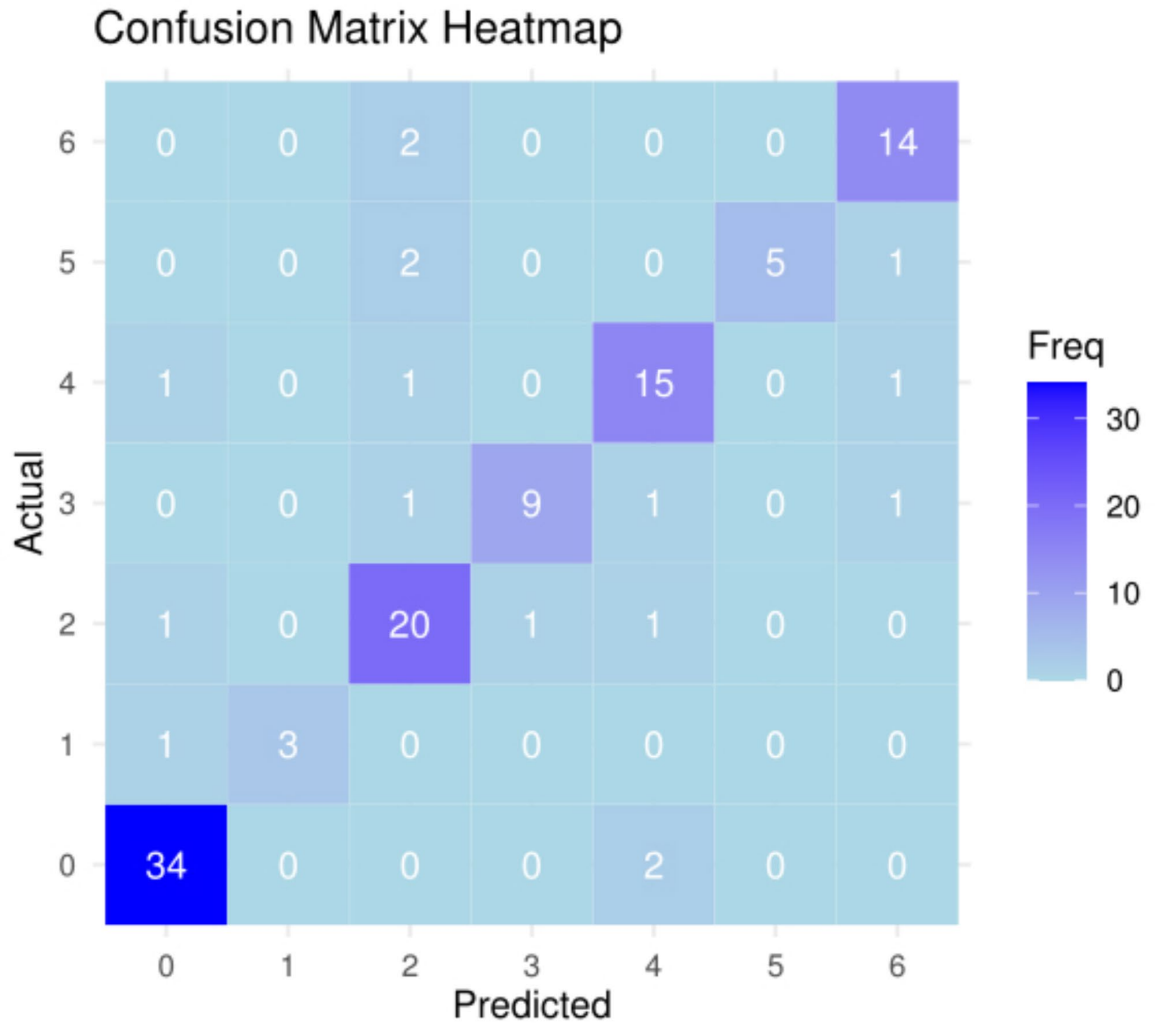
The confusion matrix heatmap. This figure presents the confusion matrix of the predicted results for the validation set in the form of a heatmap

The model’s performance was evaluated on the validation set. The confusion matrix and various classification metrics are summarized below. The Confusion Matrix Heatmap is shown in Fig. 3, using the “*ggplot2*”and “*reshape2*”packages in R.

The overall accuracy of the multiclass logistic regression model was 85.47%, with a 95% confidence interval ranging from 77.76 to 91.3%. This indicates that the model was able to correctly classify 85.47% of the samples in the validation set. The Kappa statistic was calculated as 0.8206, reflecting strong agreement between the predicted and actual classifications, and further underscoring the model’s robustness. Additionally, the model’s accuracy significantly exceeded the No Information Rate of 30.77% (*p*-value < 2.2 × 10^− 16^), confirming that the model’s performance was far better than random guessing.

### ROC curve and AUC

**Fig. 4:**
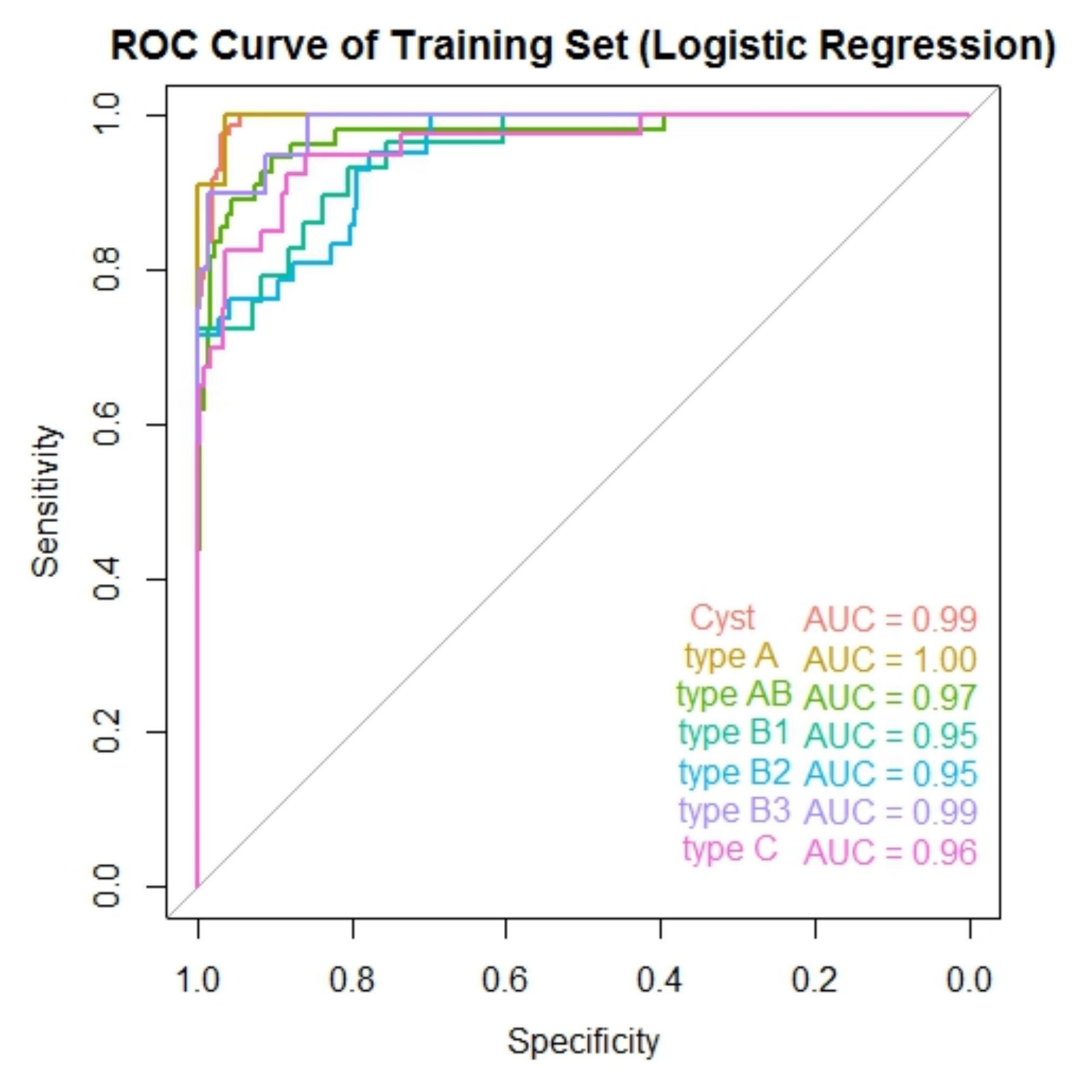
ROC curve of training set. This figure shows the ROC curves for each category in the training set, with their AUC values annotated in different colors

**Fig. 5:**
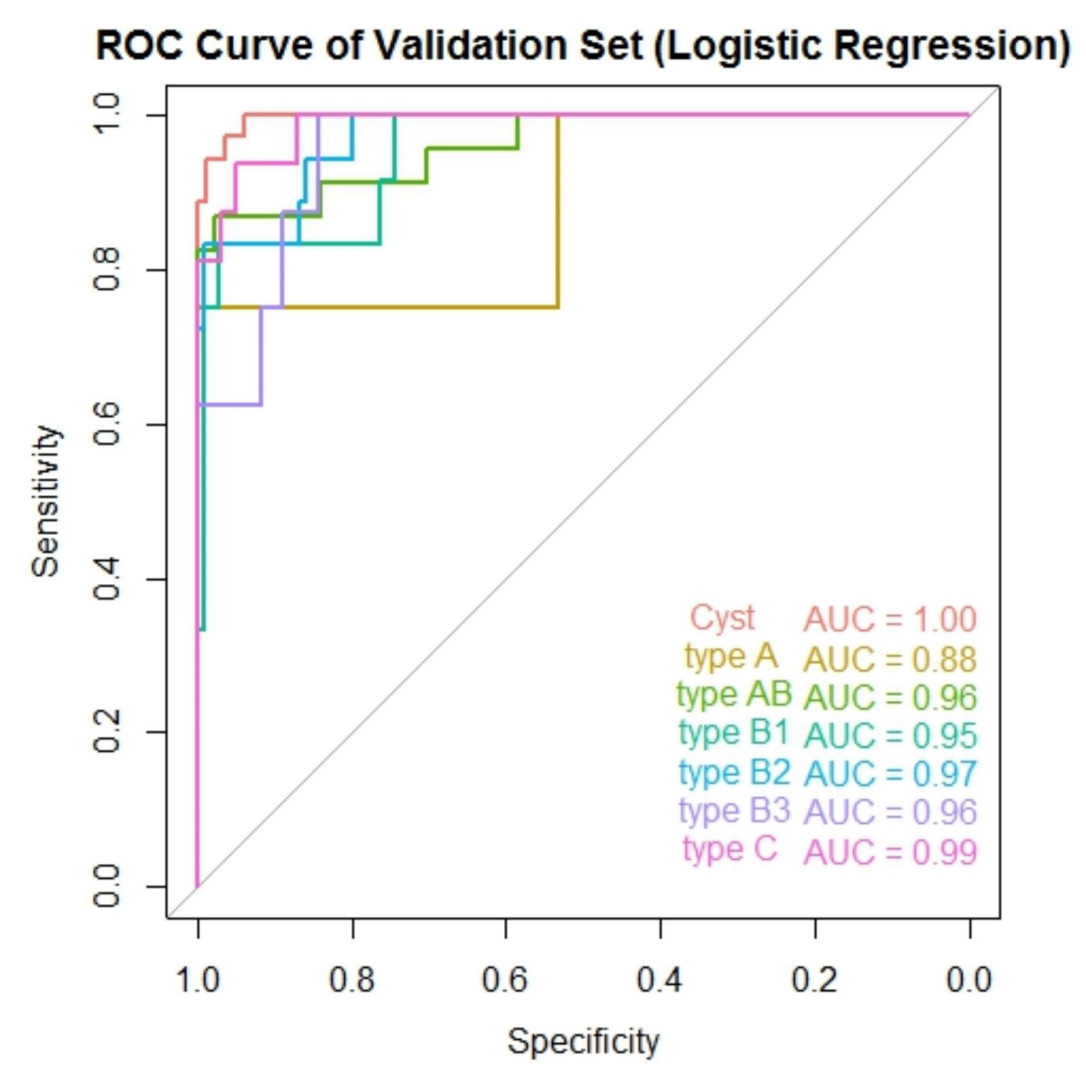
ROC curve of validation set. This figure shows the ROC curves for each category in the validation set, with their AUC values annotated in different colors

Receiver Operating Characteristic (ROC) curves for the training and validation sets, shown in Figs. 4 and 5, were plotted using the “*pROC*”package in R to evaluate the model’s ability to distinguish between the different classes. The Area Under the Curve (AUC) values for each class indicate excellent classification performance, particularly for Cyst, with an AUC of 1.00, suggesting perfect separability. Most other classes also demonstrated high AUC values, with Type AB, B2, B3, and C all achieving AUCs of 0.96 or higher, signifying strong discriminative power for these tumor types. Type A had a slightly lower AUC of 0.88, indicating good but comparatively weaker differentiation from other classes.

### Class-specific performance

Detailed performance metrics of the validation set, broken down by class, are provided in Table 2. The model exhibited high sensitivity across most classes, with Class_0 achieving a sensitivity of 94.44% and a specificity of 98.77%, demonstrating the model’s strong ability to correctly identify and distinguish samples of this class. Similarly, Class_2 showed good performance with a sensitivity of 82.61% and a specificity of 97.87%. Class_1 had a sensitivity of 75.00% and a perfect specificity of 100%, indicating that the model was highly accurate in identifying this class, though the number of samples for this class was relatively low.

**Table 2:** Performance Metrics by Class

Class	Sensitivity	Specificity	Precision	Negative Predictive Value	Balanced Accuracy
Cyst	94.44%	98.77%	97.14%	97.56%	96.60%
Type A	75.00%	100.00%	100.00%	99.12%	87.50%
Type AB	82.61%	97.87%	90.48%	95.83%	90.24%
Type B1	91.67%	96.19%	73.33%	99.02%	93.93%
Type B2	72.22%	95.96%	76.47%	95.00%	84.09%
Type B3	87.50%	100.00%	100.00%	99.09%	93.75%
Type C	81.25%	94.06%	68.42%	96.94%	87.65%

For Class_6, the sensitivity was 81.25%, with a slightly lower precision of 68.42%, suggesting that the model occasionally misclassified other classes as type C. Despite this, the Balanced Accuracy (the average of sensitivity and specificity) for Class_6 was still relatively high at 87.65%, indicating that the model’s overall ability to distinguish this class remained reasonable.

### Summary of metrics

In terms of Positive Predictive Value, most classes performed well, with Class_0 achieving 97.14% and Class_5 achieving a perfect precision of 100%. The Negative Predictive Value was similarly strong across classes, further demonstrating the model’s overall effectiveness in identifying and excluding specific types of thymic tumors.

Balanced Accuracy, which accounts for both sensitivity and specificity, was generally high across all classes, with Class_3 reaching 93.93% and Class_0 showing the highest balanced accuracy at 96.60%. The lower precision observed in Class_6 suggests that the model could be improved in distinguishing this class from other subtypes.

## Discussion

In this study, we developed a multiclass classification model to predict various TMLs types, including thymic cysts and different subtypes of thymomas, using normalized radiomic features and age. The KNN-based feature extraction followed by logistic regression demonstrated high predictive performance across multiple classes. Our model achieved an overall accuracy of 85.47% on the validation set, with a Kappa value of 0.8206, indicating a strong agreement between the predicted and actual classifications. The ROC curves further confirmed the robustness of the model, with AUC values ranging from 0.88 to 1.00 across different classes.

### Clinical impact

The predictive accuracy observed in our study is comparable to previous research on radiomic analyses of TMLs. However, it is important to note that earlier studies have primarily focused on distinguishing between cystic and solid lesions or differentiating benign from malignant solid tumors, often overlooking the more detailed subclassifications of TMLs [14–16]. In contrast, our study delves into these finer subclassifications, which provides more precise differentiation between thymic cysts and thymic epithelial tumors, offering significant clinical value.

Currently, thymectomy can be performed using various techniques, including partial thymectomy, complete thymectomy, and extended resection of the anterior mediastinum [7–9]. Surgical approaches may range from open surgery to robotic-assisted procedures, video-assisted thoracoscopic surgery, and thymectomy with sternal suspension [17–19]. As such, the accurate differentiation of TMLs could significantly aid in surgical planning, enabling more personalized treatment strategies for patients.

While the model cannot yet directly guide clinical treatment decisions, its high predictive accuracy suggests that radiomic features could serve as a valuable adjunct to non-invasive diagnosis. Our study provides reliable support for the diagnosis of thymic cysts and thymomas and holds potential to assist in clinical decision-making in the future. Future research could broaden the scope of included cases to enhance the model’s applicability across different thymic diseases and improve its clinical utility.

### Clever application of the algorithm

Within this context, our study takes a significant step forward by offering a more detailed differentiation among various categories of TMLs, albeit this naturally introduces additional complexity into the data analysis. This increased complexity presented significant challenges, especially in identifying irregular decision boundaries between different tumor types. In earlier stages of our research, we explored several alternative methods, including random forest classifiers, Mahalanobis distance classifiers, and neural network models. Despite these efforts, none of these approaches produced satisfactory results, as their accuracy did not exceed 50%. As such, we will not elaborate on these methods here.

In contrast, the KNN algorithm proved particularly effective in capturing the local characteristics of the data, allowing for better performance in classification. The strength of this algorithm lies in its ability to precisely model the subtle variations between different types of thymic lesions, which might have been missed by more complex parametric models [20]. In this study, we selected the KNN algorithm with K = 1 for classification and achieved the best performance. We believe this is due to the high separability of different categories in the local distribution of the feature space, where neighboring samples often belong to the same category. While smaller K values are generally considered prone to overfitting, in our dataset, the lower noise level and clear feature separability allowed the smaller K value to yield better classification results.

Another advantage is the inclusion of multiclass logistic regression with cross-validation, which reduces the possibility of overfitting and ensures robust generalization to unseen data [21]. Both 6-fold cross-validation and evaluation on an independent test set showed no obvious signs of overfitting, indicating that the model with K = 1 can accurately capture the local structure of the data.

### Limitations

There are some limitations in this study.

#### Limited scope of inclusion

This study did not include all anterior mediastinal tumors, such as lymphomas, germ cell tumors, neuroendocrine neoplasms, and thymic hyperplasia. These tumor types were excluded primarily due to the insufficient number of cases in the available dataset, which could have compromised the reliability of the model’s training and validation. While thymic cysts and thymomas, including types A, AB, B1, B2, B3, and C, represent the most common and clinically significant anterior mediastinal masses, the exclusion of those less common tumor types limits the generalizability of our findings to the broader spectrum of anterior mediastinal tumors.

#### Sample size

Although the sample size of this study is moderate at 400 participants, it could still be expanded to improve the generalizability of the results across diverse populations. Furthermore, while our model showed high accuracy in distinguishing between thymic cysts and various thymoma subtypes, there was a slight decrease in sensitivity and specificity for certain classes, such as Class_4 (type B2 thymoma), indicating that further refinement of feature selection or the inclusion of additional clinical parameters may be needed.

#### Feature importance analysis

As the KNN algorithm is an instance-based algorithm, it does not naturally provide feature importance like models such as decision trees or random forests. In KNN, the classification result is determined by the voting of the K nearest neighbors to the target sample, based on the smallest distance. Therefore, KNN does not generate clear feature importance scores. This limits our in-depth analysis and understanding of these radiomic features, particularly when assessing the contribution of features to model performance. The clinical significance of the results could be further explored, especially regarding the interpretability of the machine learning model [22]. While the high predictive accuracy is promising, understanding how the model arrives at specific decisions is crucial for clinical adoption. Radiomic features, although useful, require validation in clinical contexts to ensure they reflect meaningful biological processes.

#### Relationship between myasthenia gravis and thymoma

The relationship between myasthenia gravis and thymoma in clinical practice is complex, as not all myasthenia gravis patients have thymoma, nor do all thymoma patients develop myasthenia gravis. However, recent spatial transcriptomics analyses have elucidated a specialized medulla niche within myasthenia gravis-associated thymomas that supports germinal center responses, offering insights into the microenvironmental factors potentially linking these two conditions [23]. Considering that myasthenia gravis may involve more complex pathophysiological mechanisms, we decided not to include this issue in the scope of our study [24]. Although some cases in this study did indeed have myasthenia gravis, we did not differentiate these participants separately. While this limitation does not affect the construction and validation of the model, it is certainly a new direction worth exploring.

### Future directions

Future research could focus on several areas for further exploration and improvement: (a) expanding the study to include a more comprehensive range of anterior mediastinal tumors, which would provide a more holistic diagnostic tool and offer greater clinical utility; (b) increasing the sample size, especially including cases from diverse demographic and clinical backgrounds, to enhance the model’s generalizability across different populations; (c) enhancing model interpretability to help clinicians understand which features drive the model’s predictions, thereby improving clinical decision support and fostering trust and adoption; (d) conducting prospective validation in clinical settings, including real-world scenarios, to verify the model’s predictive performance; and (e) exploring the relationship between myasthenia gravis and thymoma, although this study did not analyze myasthenia gravis.

## Conclusions

In conclusion, the multiclass logistic regression model developed in this study demonstrated strong classification performance across most thymic tumor types, with high accuracy, specificity, and sensitivity. This study provides an effective tool for the classification of thymic tumors, offering potential clinical applications in the differentiation of tumor types. It can serve as a valuable diagnostic aid for clinicians, helping to optimize treatment planning.

## Electronic supplementary material

Below is the link to the electronic supplementary material.

Supplementary Material 1

Supplementary Material 2

Supplementary Material 3

Supplementary Material 4

Supplementary Material 5

Supplementary Material 6

Supplementary Material 7

## Data Availability

All data generated or analyzed and R code utilized during this study are included in this published article and its supplementary information files.

## List of Abbreviations

TMLs: Thymic mass lesions
CT: Chest computed tomography
ROIs: Regions of interest
DICOM: Digital Imaging and Communications in Medicine
e.g.: Exempli gratia
KNN: K-Nearest Neighbors
ROC: Receiver Operating Characteristic
AUC: Area Under the Curve
